# An Adipose-Derived Injectable Sustained-Release Collagen Scaffold of Adipokines Prepared Through a Fast Mechanical Processing Technique for Preventing Skin Photoaging in Mice

**DOI:** 10.3389/fcell.2021.722427

**Published:** 2021-09-24

**Authors:** Xiaoxuan Jin, Yuchen Zhang, Xiangdong Zhang, Yibao Li, Mimi Xu, Kaiyang Liu, Jiangjiang Ru, Chijuan Ma, Yao Yao, Yunfan He, Jianhua Gao

**Affiliations:** Department of Plastic Surgery, Nanfang Hospital, Southern Medical University, Guangzhou, China

**Keywords:** skin photoaging, adipokines, extracellular matrix, sustained-release, skin filling, adipose-derived product, adipose collagen concentrate

## Abstract

Ultraviolet A (UVA) radiation is the major contributor to skin photoaging, associated with increased collagen degradation and reactive oxygen species (ROS) expression. Adipokines have been proven as promising therapeutic agents for skin photoaging. However, adipokine therapy is generally limited by the short *in vivo* release duration and biological instability. Therefore, developing a treatment that provides a sustained release of adipokines and enhanced therapeutic effects is desirable. In this study, we developed a novel mechanical processing technique to extract adipose tissue-derived ECM components, named the “adipose collagen fragment” (ACF). The physical characterization, injectability, collagen components, residual DNA/RNA and adipokine release pattern of ACF were identified *in vitro*. L929 cells were treated with ACF or phosphate-buffered saline for 24 h after UVA irradiation *in vitro*. The expression of senescence-associated xβ-galactosidase (SA-β-gal), ROS and antioxidase were investigated. Then, we evaluated its therapeutic efficacy by injecting ACF and phosphate-buffered saline, as a control, into the dermis of photoaging nude mice and harvesting skin samples at weeks 1, 2, and 4 after treatment for assessment. The content of adipokines released from ACF was identified *in vivo*. The collagen synthesis and collagen degradation in ACF implants were evaluated by immune staining. Dermal thickness, fibroblast expression, collagen synthesis, ROS level, antioxidase expression, capillary density, and apoptotic cell number were evaluated by histological assessment, immune staining, and polymerase chain reaction in the skin samples. We demonstrated that ACF is the concentrated adipose extracellular matrix collagen fragment without viable cells and can be injected through fine needles. The lower expression of SA-β-gal, ROS and higher expression of antioxidase were observed in the ACF-treated group. ACF undergoes collagen degradation and promotes neocollagen synthesis in ACF implants. Meanwhile, ACF serves as a sustained-release system of adipokines and exhibits a significantly higher therapeutic effect on mouse skin photoaging by enhancing angiogenesis, antioxidant abilities, antiapoptotic activities, and collagen synthesis through sustainedly releasing adipokines. To sum up, ACF is an adipokines-enriched, sustained-release extracellular matrix collagen scaffold that can prevent UVA-induced skin photoaging in mice. ACF may serve as a novel autologous skin filler for skin rejuvenation applications in the clinic.

## Introduction

Photoaging attributed to chronic sun exposure [ultraviolet A (UVA) radiation] is a major contributor to skin aging (Gilchrest, 2013; Wang et al., 2014; Kammeyer and Luiten, 2015; Liu and Zhang, 2015). UVA exposure increases reactive oxygen species (ROS) production through oxidative metabolism in skin cells (Fabi and Sundaram, 2014; Bosch et al., 2015). ROS expression is thought to be a key mediator of fibroblast viability by introducing intracellular DNA damage and protein inactivation (Rinnerthaler et al., 2015). Impairments in dermal fibroblasts result in decreased collagen production and remodeling, leading to thin, saggy, and structurally weakened skin (Xia et al., 2015; Purohit et al., 2016). Therefore, more effective strategies are needed to reduce oxidative stress levels and attenuate UVA-induced cell death to prevent skin photoaging.

The topical application of antioxidants and stem cells has been proven to be effective in preventing skin photoaging. However, their use in clinics has been limited by poor permeability, healthcare regulatory issues, poor survival of administered cells, and the risk of biological contamination (Masaki, 2010; Sato et al., 2019; Yeager and Lim, 2019). The mechanisms underlying stem cell therapy have been largely attributed to cellular paracrine cytokines (Kim et al., 2009; Altman et al., 2010; Xu et al., 2014). These cytokines act on skin dermal cells to improve skin quality and resist skin aging (Hodgkinson et al., 2016). Recently, studies reported that subcutaneous adipose tissue, located beneath the dermal layer, is closely involved in regulating skin elasticity and contributes to skin physiology (Ezure and Amano, 2010; Lee et al., 2020). Importantly, subcutaneous adipose tissue influences dermal conditions through the secretion of various bioactive substances, termed adipokines (Halberg et al., 2008; Kim et al., 2016). Thus, adipokines appear to be ideal therapeutic agents for preventing skin photoaging. Recently, our research team extracted adipokines, namely adipose liquid extract, from adipose tissue using a purely mechanical method (Yu et al., 2018; Deng et al., 2019; He et al., 2019; Cai et al., 2020). Adipose liquid extract contains 1,742 bioactive components and has been found to have a therapeutic effect on wound healing and skin aging by improving angiogenesis, cell viability, collagen synthesis, and attenuating oxidative stress. Nevertheless, given that skin aging is an inevitable and continuous physiological process (Gal and Pu, 2020; Gu et al., 2020), and that the *in vivo* release duration and biological stability of adipokines are unsatisfactory, a vehicle that can provide a sustained prolonged release as well as maintaining high stability of adipokines with enhanced therapeutic efficacy is desirable.

Adipose tissue is mainly composed of mature adipocytes, stromal vascular fraction, and the extracellular matrix (ECM) (Mori et al., 2014). The ECM provides natural binding domains to store adipokines secreted by stromal vascular fraction (SVF) cells (van Dongen et al., 2019). These adipokines are generally released from ECM scaffolds when exposed to the appropriate stimuli (Bhardwaj et al., 2000; Kanematsu et al., 2004; Hynes, 2009; Choi et al., 2010). Therefore, we hypothesized that adipose-derived ECM components could be extracted and serve as a sustained-release scaffold of adipokines for the treatment of skin photoaging.

To verify this hypothesis, we developed a novel mechanical processing technique to extract adipose tissue-derived ECM components, named the “adipose collagen fragment” (ACF). The macrography, physical characterization, injectability, residual DNA/RNA, and protein components in ACF were evaluated, and the sustained-release properties of ACF were measured *in vitro* and vivo. Moreover, the therapeutic effect of ACF on UVA-induced photoaging in cells and mice was evaluated *in vitro* and *in vivo* using phosphate buffered saline (PBS) as the control.

## Materials and Methods

### Human Adipose Tissues Harvest and Adipose Collagen Fragment Preparation

**FIGURE 1:**
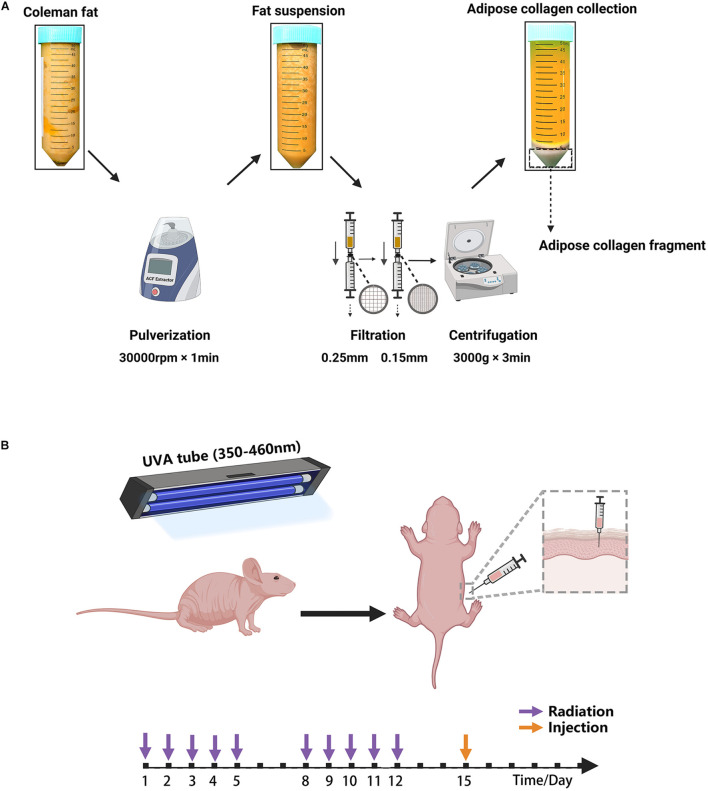
**(A)** Schematic overview of ACF preparation. **(B)** Illustration of the animal experiment. The mouse was irradiated by a UVA tube for 2 weeks and then injected with ACF and PBS.

Human adipose tissues in the abdominal regions were obtained from female individuals who underwent liposuction in the Department of Plastic and Reconstruction Surgery, Nanfang Hospital. All clinical procedures performed in this study were approved by the Nanfang Hospital Ethics Committee and all patients provided written informed consent (K2019018). ACF was prepared from fresh lipoaspirates. First, the lipoaspirate was centrifuged at 1,200 × g for 3 min to generate Coleman fat. After washing twice with sterilized saline and homogenizing for 60 s in an ACF extractor (Specially made; Shanghai Tiangong Instruments Co., Ltd., Shanghai, People’s Republic of China), the fat suspension was transferred into 20 ml syringes and then filtered consecutively using unidirectional filters containing a sterilized round stainless-steel filter screen with a 0.25- or 0.15-mm sized mesh (Supplementary Figure 1). Then, the fat suspension was centrifuged at 3,000 × g for 3 min, and the solid portion at the bottom was collected as ACF (Figure 1A and Supplementary Video 1).

### Macrography Evaluation and Collagen Component Analysis of Adipose Collagen Fragment

ACF was injected through 31-gauge needles to verify the injectability of ACF. For physical characterization evaluation, ACF, Coleman fat and water were put into the glass bottles and the bottles were tilted 45 degrees. For histological evaluation, ACF and Coleman fat were loaded onto a glass slide, and the samples were picked up with tweezers and observed under a light microscope. ACF and standard Coleman fat samples were fixed with 4% paraformaldehyde, embedded in paraffin, and sliced into 5-μm thick sections. Masson staining was performed to assess collagen components. Immunohistochemical staining was performed using antibodies against COL I (ab34710, Abcam, Cambridge, United Kingdom), COL IV (ab6586, Abcam, Cambridge, United Kingdom), and laminin (ab11575, Abcam, Cambridge, United Kingdom).

### Live/Dead Cell Staining and Explant Culture

The cell viability of Coleman fat and ACF was evaluated using live/dead staining. Briefly, 1 ml of PBS supplemented with 2 μl of Calcein-AM (1 mg/ml) (AnaSpec, Fremont, Calif.) and 2 μl of PI (1 mg/ml) (Sigma-Aldrich, St. Louis, MO, United States) was added to 1 ml of Coleman fat or ACF samples. After incubating for 10 min at 37°C, the samples were observed under a fluorescence microscope (BX 51, Olympus, Japan). The experiment was independently repeated three times.

The explant culture was performed according to a standard protocol (Sineh et al., 2020). Briefly, the Coleman fat and ACF were evenly distributed on the surface of 100 mm culture dishes (1 ml/dish). The samples were cultured in complete growth medium (HUXMD-90011, Cyagen, China) at 37°C with 5% humidified CO_2_ for 5 days. Then, the outgrown cells from explants were observed under a light microscope (GX53, Olympus, Japan). The experiment was independently repeated three times.

### Proteomic Mass Spectrometry and Bioinformatics Analysis of Adipose Collagen Fragment

The Trypsin was used to digest the ACF samples for LC-MS/MS analysis according to a previous protocol (Wisìniewski et al., 2010). LC-MS/MS analysis was performed using a nano LC system (DIONEX Thermo Fisher Scientific). The tryptic peptides were fractionated and subjected to reversed-phase liquid chromatography. Peptide samples were separated on a self-packed column (Thermo Fisher Scientific, Acclaim PepMap RSLC 50 μm × 15 cm, nanoviper, P/N164943) at a flow rate of 300 nL/min, according to a previous protocol (Hauck et al., 2010). Label-free quantification (LFQ) was performed as previously described (Luber et al., 2010). The resulting data for LFQ were processed using the MaxQuant program (version 1.5.3.1), and Andromeda was used to match MS/MS spectra as a database search engine according to the human database (Cox et al., 2011).

Gene Ontology (GO) database^1^ was used to perform functional annotation analysis, including cell component, molecular function, and biological process (Gotz et al., 2008). Protein location information of all identified proteins in ACF was obtained using Ingenuity Pathway Analysis analysis through a web database.^2^ Proteins participated in diverse collagen biological process were identified. Collagen types of ACF were classified according to the protein identification list. Proteins related to angiogenesis, antioxidant ability, cell proliferation, and apoptosis were identified and summarized.

### DNA/RNA Content Measurement

Coleman fat and ACF samples were used to assess presence of DNA/RNA. Briefly, the solution containing 10% SDS or guanidinium isothiocyanate (Abcam, Cambridge, United Kingdom) was added to 200 mg of samples to extract DNA/RNA from Coleman fat and ACF. DNA/RNA was quantified with the use of a spectro-photometer at the wavelength of 230, 260, or 280 nm. The experiment was independently repeated three times.

### *In vitro* Release of Adipokines From Adipose Collagen Fragment

The ACF were cultured with Dulbecco’s modified Eagle’s medium (Thermo Fisher Scientific, Foster City, CA, United States) in 100 mm plate dishes and maintained at 37°C with 5% CO_2_. DMEM (9 ml) was added to 1 ml of ACF to each dish. The culture medium was replaced on days 2 and 6 and collected on days 1, 3, and 7. Protein concentrations of the culture media were measured using a BCA protein assay kit to determine the total amount of released adipokines. To identify the amount of released fibroblast growth factor (FGF), vascular endothelial growth factor (VEGF), and adiponectin, protein quantification of the culture medium was carried out using ELISA kits (R&D Systems, Minneapolis, MN, United States). The experiment was repeated three times.

### Cell Culture and Treatments

Murine L929 fibroblasts (Keyi Biotechnology Company, Hangzhou, China) were grown in monolayer in Dulbecco’s modified Eagle’s medium, supplemented with 10% Fetal bovine serum (Thermo Fisher Scientific Inc., Waltham, MA, United States), and maintained at 37°C with 5% CO_2_. Senescent L929 cells were obtained by exposure to UVA light (100 mJ/cm^2^). Then the cells were incubated with 0.5 ml PBS or ACF, respectively, for 24 h in basic medium without FBS at 37°C containing 5% CO_2_.

### Cell Evaluation *in vitro*

To detect senescence cells, senescence-associated β-galactosidase (SA-β-gal) staining was carried out 24 h post-treatment with a SA-β-gal staining kit according to the manufacturer’s instructions (Danvers, MA, United States). Intracellular ROS levels were measured by the DCFH2-DA staining kit (S0063, Beyotime, China). The cells were then observed under a microscope (BX 51, Olympus, Japan; LSM 980, Zeiss Axioscope, Oberkochen, Germany). Qualification analyses of ROS levels were assessed using Image J software.

### Animals and Skin Photoaging Model

All experiments were approved by the Nanfang Hospital Animal Ethics Committee Laboratory and were conducted according to the National Health and Medical Research Council of China guidelines. In total, 18 6-week-old female BALB/c nude mice were obtained from the Southern Medical University and maintained in a regulated environment (22 ± 2°C) with a 12 h light/dark cycle at the Animal Experiment Center of Nanfang Hospital, and were fed according to the specific pathogen-free animal criteria. A mouse model of photoaging was used as previously described (Zhou et al., 2020; Figure 1B). Briefly, 40 W UVA tubes (wavelength range: 350–460 nm; 13 × 10^2^ μW/cm^2^; Sigma-Aldrich, Shanghai, China) were used to irradiate the dorsal skin. The mice were irradiated for 2 weeks, once daily, for 5 days a week. We followed an irradiation approach in which the duration of UVA irradiation was 1 h on day 1, after which the duration of UVA irradiation was increased by 1 h per day up to 5 h. The mice were then irradiated for 5 h daily from days 8–12. The total irradiation intensity of the UVA was approximately 172 J/cm^2^.

### Adipose Collagen Fragment Labeling and Animal Experiments

The collagen components of ACF were labeled immediately after preparation according to a previously described protocol (Correa-Gallegos et al., 2019). Briefly, 3 ml of ACF were incubated with 100 μM Alexa Fluor 647 NHS Ester (A20006, Thermo Fisher Scientific, Foster City, CA, United States) for 1 h at 25°C, followed by three washes with PBS.

The ACF after labeling was injected immediately into the mouse skin of the left dorsa (9 spots/side, 0.01 ml/spot, total 0.09 ml), with a PBS injection into the right side serving as the control (9 spots/side, 0.01 ml/spot, total 0.09 ml). Animals were sacrificed (*n* = 6 mice per time point), and skin samples were harvested for further analyses on weeks 1, 2, and 4 postoperatively (*n* = 6/time point). One-half of the skin samples were fixed with paraformaldehyde, and the other half of the skin samples were immediately stored at -80°C for further experiments.

### Western Blot Analysis

The expression of cellular catalase, SOD-1 and GPX-1 were measured using Western blot analysis according to the standard protocol. Human-derived proteins (adiponectin, VEGF and FGF) were detected by western blot analysis using the specific antibodies according to the standard protocol. Total samples lysates were prepared using M-PER Mammalian protein extraction reagent (Thermo Fisher Scientific). Protein concentrations were determined using a BCA Protein Assay Kit (Beyotime, China). Membranes were incubated with primary antibodies: anti-catalase (ab16731, Abcam), anti-SOD-1 (ab13498, Abcam), anti-GPX-1 (ab108427, Abcam), anti-adiponectin (ab75989; Abcam), anti-VEGF (ab183100; Abcam), and anti-FGF (ab179455; Abcam). After incubation with secondary antibodies, the detection was performed with Western Breeze Chemiluminescent Detection Kit (Thermo Fisher Scientific). β-actin (ab6276, ab264083; Abcam) served as an internal control. Quantitative analysis of the protein amount using Image J software.

### Histological Assessment and Immune Staining of Skin Samples

Mouse skin samples were embedded in paraffin and sliced into 5-μm thick sections. Masson staining was performed to assess collagen content in the dermis and ACF implants. The expression of fibroblasts and newly formed collagen in the dermis and ACF implants was evaluated using antibodies against vimentin (ab92547, Abcam, Cambridge, United Kingdom) and procollagen (ab64409, Abcam, Cambridge, United Kingdom), followed by secondary antibodies. Angiogenesis was evaluated using antibodies against CD31 antibody (1:25, ab28364, Abcam, Cambridge, United Kingdom), followed by secondary antibodies. A TUNEL staining kit (Roche Molecular Biochemicals, Mannheim, Germany) was used for detecting apoptotic cells in skin samples according to the manufacturer’s protocol. ROS levels were measured by the DCFH2-DA staining kit (S0063, Beyotime, China). The expression of antioxidant enzymes in skin samples was evaluated using antibodies against superoxide dismutase-1 (SOD-1; PB0453, Boster, China), catalase (PB0971, Boster, China), and glutathione peroxidase-1 (GPX-1; PB9203, Boster, China), followed by secondary antibodies. The sections were then observed under a microscope (BX 51, Olympus, Japan; LSM 980, Zeiss Axioscope, Oberkochen, Germany). Qualification analyses of dermal thickness, fibroblasts, neocollagen, capillary density, apoptotic cells, ROS levels, and antioxidant enzymes were assessed using Image J software.

### Quantitative Reverse Transcription PCR

Total RNA was extracted and reverse transcribed into cDNA using a reverse transcription kit (Thermo Fisher Scientific, Foster City, CA, United States). cDNA was amplified and taken as a template. qRT-PCR was performed on QuantStudio Real-Time PCR Systems (Applied Biosystems, United States). The relative mRNA expression was calculated by the 2^–ΔΔCt^ method, with β-actin as the reference gene. Primer sequences were used as follows: SOD-1, forward 5-GGTTCCACGTCCATCAGT-3 and reverse 5-ACATTGCCCAGGTCTCC-3; catalase, forward 5-GAAGGCTTGCTCAGGAAGAT-3 and reverse 5-TGCCAACT GGTATAAGAGGGTA-3; GPX-1, forward 5-ATCAGTTCGG ACACCAGGA-3 and reverse 5-TCTCACCATTCACTTCGCA-3; β-actin, forward 5-GAGGTATCCTGACCCTGAAGTA-3 and reverse 5-CACACGCAGCTCATTGTAGA-3′.

### Statistical Analysis

All data are expressed as mean ± SD. Statistical analyses were performed using SPSS software (version 26.0; IBM Corp., Armonk, NY, United States). An unpaired *t*-test was used to compare the two groups at a single time point. Statistical significance was set at *p* < 0.05.

## Results

### Adipose Collagen Fragment Is an Adipose Extracellular Matrix Fragment Without Viable Cells

**FIGURE 2:**
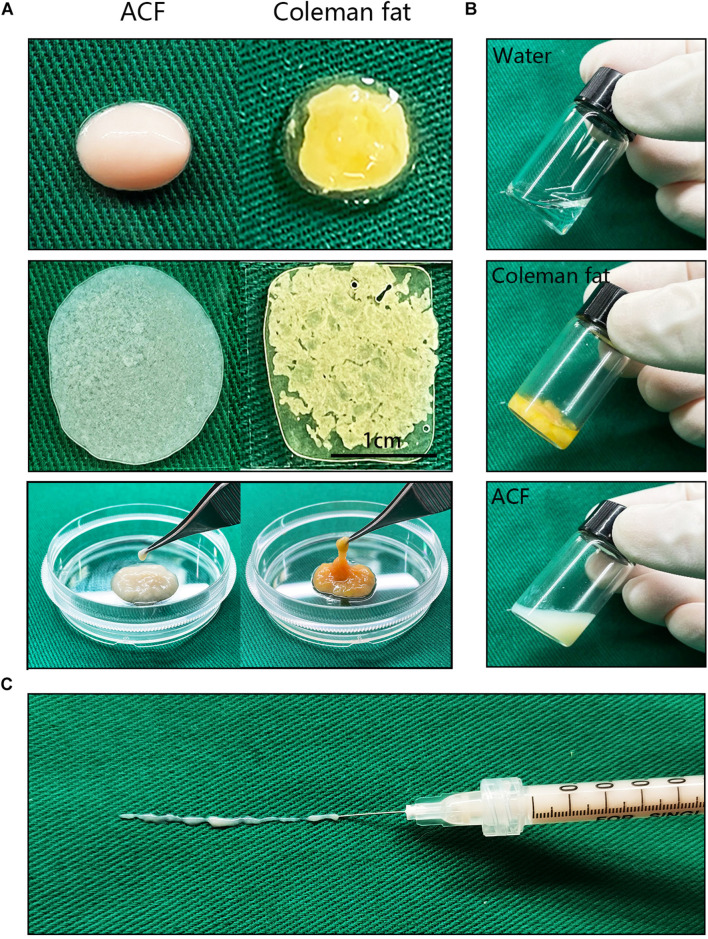
Macroscopic observation and injectability of ACF. **(A)** Macroscopic observation of Coleman fat and ACF. ACF is a pinkish-white, homogeneous, and viscous substance with a smooth texture. Scale bar = 1 cm. **(B)** ACF is a viscous semifluid. Coleman fat can hardly tilt, the water can tilt immediately, and ACF can tilt completely after 54.72 ± 0.23 s. **(C)** ACF could be injected through 31-gauge sharp needles.

**FIGURE 3:**
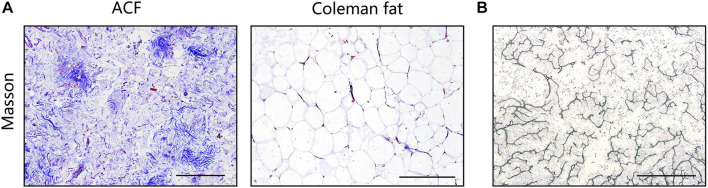
Histological evaluation of Coleman fat and ACF. **(A)** Masson staining of Coleman fat and ACF. Collagen was expressed in the entire field of view in ACF, while the collagen components in Coleman fat can only be detected in the surrounding adipocytes. Scale bar = 200 μm. **(B)** Scattered, stripe-like collagen fragments in ACF were observed in the bright-field image. Scale bar = 1 cm.

**FIGURE 4:**
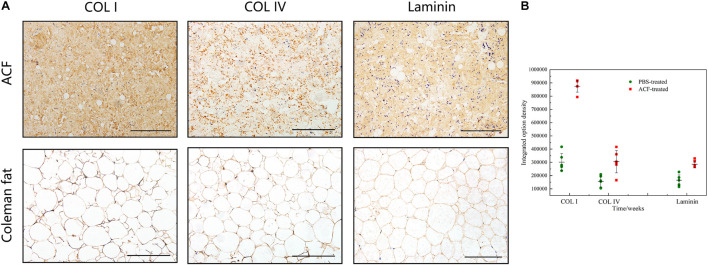
Collagen components assessment of ACF. **(A)** Immunohistochemical staining of COL I, COL IV, and laminin in ACF. ACF is composed of collagen fragments. COL I, COL IV, and laminin expression in ACF can be detected throughout the entire field of view. **(B)** Semi-quantitative analysis of collagen I, collagen IV, and laminin in ACF. Scale bar = 100 μm. P (collagen I) = 7.003E-09, F (collagen I) = 0.407; P (collagen IV) = 0.003, F (collagen IV) = 0.169; P (laminin) = 1.001E-04, F (laminin) = 0.293.

**FIGURE 5:**
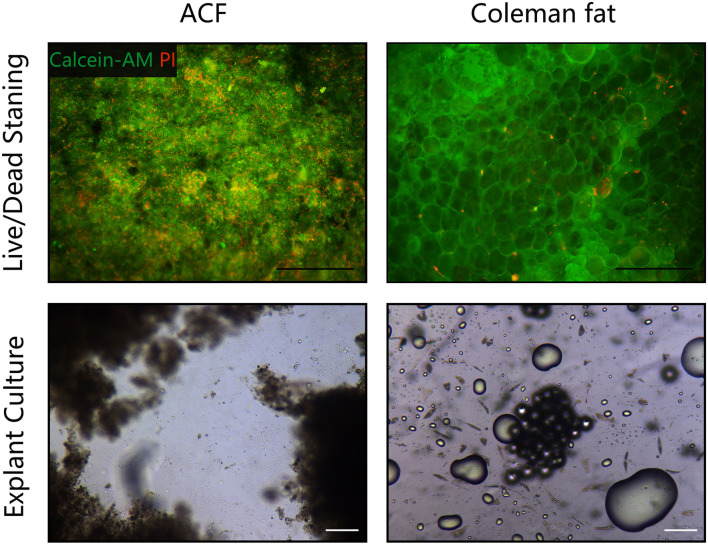
Cell viability assessment of ACF and Coleman fat. **(Above)** Live/dead staining of ACF and Coleman fat. Scale bar = 100 μm. Massive dead cells (red) were observed in the ACF, while only a few dead cells (red) were observed in Coleman fat. **(Below)** Explant culture assays of ACF and Coleman fat. No outgrown cells were observed in the ACF explant culture assays; nevertheless, many fibroblastic-like cells were detected outgrown from Coleman fat explants. Scale bar = 200 μm. The experiment was independently repeated three times.

Figure 2A shows macroscopic observations of Coleman fat and ACF. ACF is a pinkish-white, homogeneous, and viscous substance with a smooth texture. Coleman fat can be partially picked up with tweezers, while ACF cannot be picked up at all. Figure 2B shows that the Coleman fat can hardly tilt, the water can tilt immediately, and ACF can tilt completely after 54.72 ± 0.23 s. These results show that ACF is a viscous semifluid. Meanwhile, ACF can be injected through 31-gauge needles (Figure 2C). Figure 3A shows that collagen was expressed in the entire field of view in ACF, while the collagen components in Coleman fat can only be detected in the surrounding adipocytes. Figure 3B shows that ACF is composed of collagen fragments. COL I, COL IV, and laminin expression in ACF can be detected throughout the entire field of view, while these collagens can only be observed surrounding adipocytes in Coleman fat (*p* < 0.05) (Figures 4A,B). Massive dead cells (red) were observed in the ACF, while only a few dead cells (red) were observed in Coleman fat (Figure 5). No outgrown cells were observed in the ACF explant culture assays; nevertheless, many fibroblastic-like cells were detected outgrown from Coleman fat explants. Coleman fat samples contained DNA with 339.389 ng/μl and RNA with 373.667 ng/μl; ACF samples contained DNA with 349.368 ng/μl and RNA with 811.618 ng/μl.

### Adipose Collagen Fragment Is an Adipokines Sustained-Release Collagen Scaffold

**FIGURE 6:**
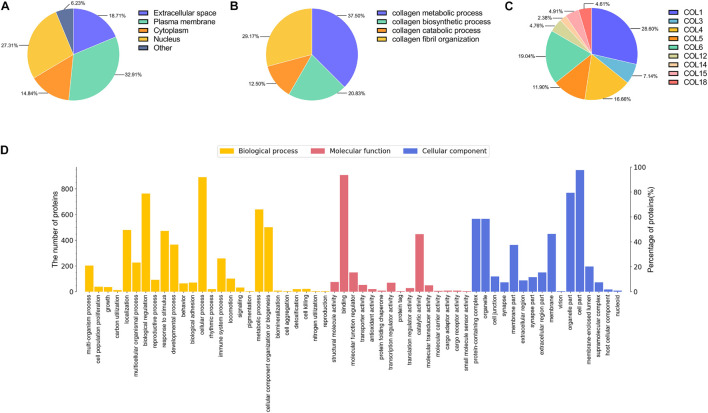
Mass spectrometry-based quantitative proteomic analysis of ACF and classification of identified proteins. **(A)** Subcellular distribution of proteins in ACF. **(B)** Distribution of proteins involved in the biological process of collagen in ACF. **(C)** Distribution of collagen types in ACF. The most abundant collagen types were type I, type VI, and type IV. **(D)** Biological processes and molecular function and cellular components categories of identified proteins in ACF.

A total of 2,555 proteins were quantified. Proteins were classified by GO annotation based on three categories: cellular components, molecular functions, and biological processes (Figure 6D). The three most abundant classes of biological processes were cellular processes, biological regulation, and metabolic processes. In addition, the molecular function analysis showed that most of the quantified proteins in ACF were classified in the classes of binding, catalytic activity, and molecular function regulators. For the result of the cellular component, the majority of ACF proteins were in the cell, organelle, and protein-containing complex GO category. Figure 6A shows that most of the quantified proteins of ACF were from the plasma membrane, followed by the nucleus, and finally from the extracellular space and cytoplasm. Figure 6B shows that most proteins participated in the collagen metabolic process and collagen fibril organization, followed by the collagen biosynthetic process and collagen catabolic process. Figure 6C describes that the most abundant collagen types were type I, type VI, and type IV. Functional annotation revealed that a great variety of proteins are involved in angiogenesis, antioxidation, cell proliferation, and apoptosis (Supplementary Tables 1, 2).

**FIGURE 7:**
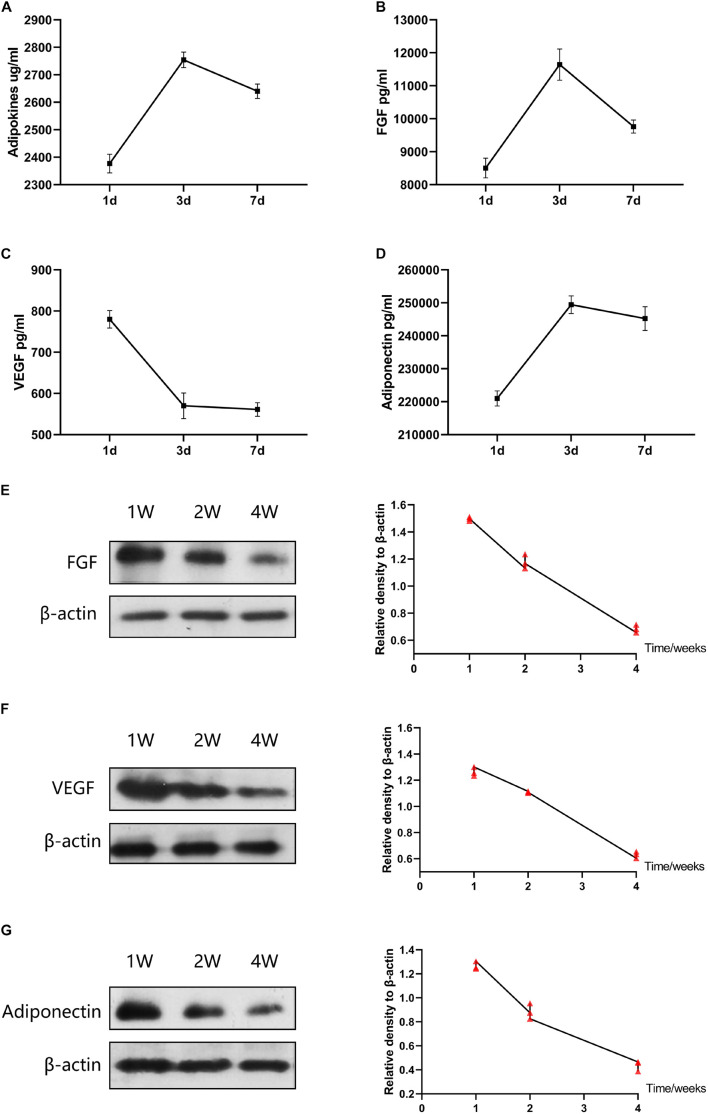
Adipokines release of ACF *in vitro* and *in vivo*. **(A–D)** Concentrations of total adipokines, FGF, VEGF, and adiponectin released from ACF on days 1, 3, and 7 *in vitro*. The experiment was independently repeated three times. **(E–G)** Western blot of FGF, VEGF, and adiponectin in skin samples at week 1, 2, and 4. The contents of FGF, VEGF, and adiponectin in skin samples were gradually decreasing over time in the 4 weeks. P (FGF at week 1–2) = 5.273E-04, F (FGF at week 1–2) = 0.143; P (FGF at week 1–4) = 1.706E-06, F (FGF at week 1–4) = 0.399; P (FGF at week 2–4) = 1.404E-04, F (FGF at week 2–4) = 0.472. P (VEGF at week 1–2) = 1.521E-03, F (VEGF at week 1–2) = 0.062; P (VEGF at week 1–4) = 1.346E-05, F (VEGF at week 1–4) = 0.701; P (VEGF at week 2–4) = 5.723E-06, F (VEGF at week 2–4) = 0.111. P (adiponectin at week 1–2) = 8.120E-04, F (adiponectin at week 1–2) = 0.417; P (adiponectin at week 1–4) = 1.224E-05, F (adiponectin at week 1–4) = 0.742; P (adiponectin at week 2–4) = 5.626E-04, F (adiponectin at week 2–4) = 0.617. *N* = 3.

Figure 7 shows the release curves of adipokines of ACF *in vitro*. Adipokines release increased over time from days 1 to 3, and reached a maximum value on day 3. Adipokines concentrations decreased over time in the subsequent 4 days (Figure 7A). The amount of FGF released from ACF increased over time from days 1 to 3, and the FGF release reached a maximum value on day 3 (Figure 7B). The release concentration of FGF decreased over the following 4 days. VEGF release reached a maximum value on day one and then decreased from days 1 to 3 (Figure 7C). The released VEGF displayed a plateaued release between days 3–7. Over the following 7 days, the concentration of VEGF decreased over time. ACF released adiponectin rapidly from days 1 to 3, and adiponectin release reached a maximum value on day 3 (Figure 7D). Adiponectin concentrations decreased over time in the subsequent 4 days. *In vivo*, the content of FGF, VEGF and adiponectin in skin samples was measured by western blot analysis (Figures 7E–G). Semi-quantification analysis demonstrated a gradually decreasing content of these factors over time in the 4 weeks.

### Adipose Collagen Fragment Prevents SA-β-Gal-Positive Cell Expression, Reduces Reactive Oxygen Species Production and Induces Antioxidant Proteins Expression in Ultraviolet A-Induced Cells

**FIGURE 8:**
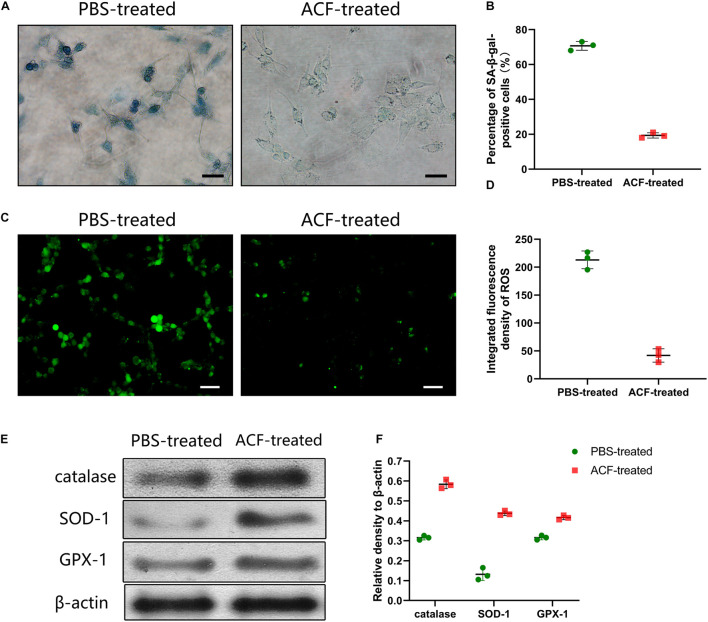
Effects evaluation of ACF with UVA-induced L929 cells *in vitro*. **(A)** The expression level of SA-β-gal-positive cells of ACF-treated and PBS-treated groups. **(B)** Semi-quantification analysis demonstrated that the number of SA-β-gal-positive cells was significantly higher in the PBS-treated group than in the ACF-treated group. *P* = 7.159E-06, *F* = 0.538. Scale bar = 150 μm. **(C)** ROS production evaluated by dihydroethidium staining of three groups. **(D)** Semi-quantitative analysis showed a higher level of ROS was observed in the PBS-treated group than in the ACF-treated group. *P* = 1.194E-04, *F* = 0.717. Scale bar = 200 μm. **(E)** Western blot analysis of catalase, SOD-1 and GPX-1 in both groups. **(F)** Semi-quantitative analysis showed the significantly higher expression of the antioxidase in ACF-treated group compared with the PBS-treated group. P (catalase) = 4.505E-05, F (catalase) = 0.405; P (SOD-1) = 8.572E-05, F (SOD-1) = 0.255; P (GPX-1) = 3.411E-04, F (GPX-1) = 0.957. *N* = 3.

SA-β-gal staining showed that the expression level of SA-β-gal-positive cells was significantly decreased in the ACF-treated group than the control group (Figure 8A). Semi-quantification analysis demonstrated that the number of SA-β-gal-positive cells was significantly higher in the PBS-treated group than in the ACF-treated group (*p* < 0.05) (Figure 8B). Figure 8C revealed a lower level of ROS in the ACF-treated group than that in the PBS-treated group (*p* < 0.05) (Figure 8D). Western blot analysis showed the significantly higher expression of catalase, SOD-1 and GPX-1 in the ACF-treated group compared with the PBS-treated group (*p* < 0.05) (Figures 8E,F).

### Adipose Collagen Fragment Undergoes Collagen Degradation and Promotes Neocollagen Synthesis in Adipose Collagen Fragment Implants

**FIGURE 9:**
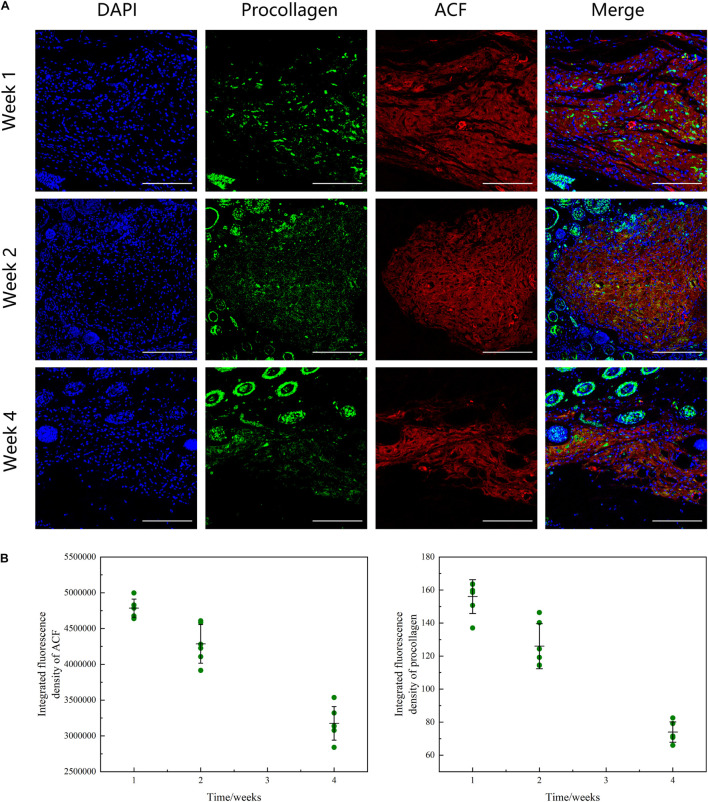
Histological evaluation of ACF *in vivo* remodeling process. **(A)** Immunofluorescence staining of AF647 labeled-ACF and procollagen. ACF underwent a degradation process from weeks 1 to 4 and procollagen expression in ACF implant was maximal at week 1 and then decreased from weeks 2 to 4. **(B)** Semi-quantitative analysis of ACF retention and procollagen expression in ACF. Scale bar = 200 μm. P (ACF retention at week 1–2) = 0.002, F (ACF retention at week 1–2) = 0.121; P (ACF retention at week 1–4) = 1.810E-05, F (ACF retention at week 1–4) = 0.767; P (ACF retention at week 2–4) = 3.990E-08, F (ACF retention at week 2–4) = 0.202. P (procollagen at week 1–2) = 0.002, F (procollagen at week 1–2) = 0.542; P (procollagen at week 1–4) = 5.720E-06, F (procollagen at week 1–4) = 0.103; P (procollagen at week 2–4) = 1.137E-08, F (procollagen at week 2–4) = 0.286.

Figure 9A shows the phenomenon of collagen degradation in ACF *in vivo*. ACF underwent a slow degradation process from weeks 1 to 2 (*p* > 0.05), followed by a fast degradation process from weeks 2 to 4 (*p* < 0.05) (Figure 9B). Procollagen expression was detected in ACF implants at all-time points. In ACF implants, the semi-quantification analysis showed that procollagen expression was maximal at week one and then decreased from weeks 2 to 4 (*p* < 0.05).

### Adipose Collagen Fragment Enhances the Dermal Thickness in the Mouse Dermis and Promotes Fibroblast Expression in Adipose Collagen Fragment Implants and the Mouse Dermis

**FIGURE 10:**
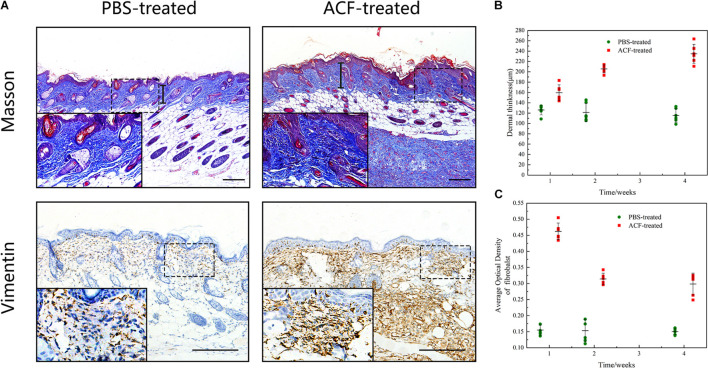
Masson staining and the fibroblast expression of mouse skin samples. **(A)** Dermal thickness and the fibroblast expression of both groups at week 1. The arrows indicate the dermal portion of the skin. The significantly higher level of dermal thickness and fibroblast expression was detected in the ACF-treated group. **(B)** Semi-quantitative analysis of the dermal thickness of both groups at all-time points. P (week 1) = 0.001, F (week 1) = 0.309; P (week 2) = 7.438E-07, F (week 2) = 0.098; P (week 4) = 1.50E-07, F (week 4) = 0.472. **(C)** Semi-quantitative analysis of the fibroblast expression in the dermis of both groups at all-time points. P (week 1) = 2.223E-10, F (week 1) = 0.300; P (week 2) = 1.400E-06, F (week 2) = 0.160; P (week 4) = 1.063E-06, F (week 4) = 0.297. Scale bar = 200 μm.

Masson staining showed a significantly higher level of dermal thickness and collagen expression in the ACF-treated group at week 1, compared to the control group (Figure 10A). Semi-quantification analysis demonstrated that the dermal thickness of the ACF-treated group was higher than that in the control group at each time point (*p* < 0.05) (Figure 10B). Immunohistochemical staining showed that fibroblasts were observed in mouse skin and ACF implants at week 1 (Figure 10A). Semi-quantification analysis demonstrated that the expression level of fibroblasts in the dermis of the ACF-treated group was maximal at week 1 and decreased from weeks 2 to 4, and was higher than that in the control group at each time point (*p* < 0.05) (Figure 10B).

### Adipose Collagen Fragment Reduces Reactive Oxygen Species Production and Induces Antioxidant Proteins Expression in the Mouse Dermis

**FIGURE 11:**
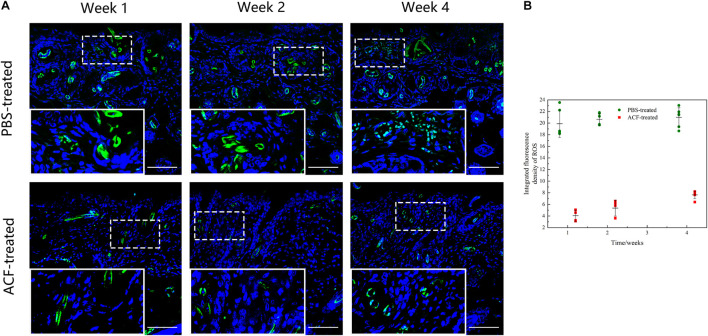
ROS production of mouse skin samples of ACF-treated group and control group. **(A)** ROS production evaluated by dihydroethidium staining of both groups. A higher level of ROS was observed in the control group than in the ACF-treated group at each time point. **(B)** Semi-quantitative analysis of the ROS production in the dermis of both groups. P (week 1) = 3.081E-08, F (week 1) = 0.070; P (week 2) = 9.081E-10, F (week 2) = 0.560; P (week 4) = 4.837E-09, F (week 4) = 4.836E-09. Scale bar = 200 μm.

**FIGURE 12:**
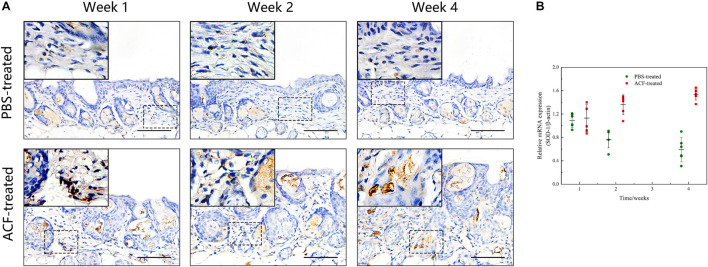
The expression of SOD-1 of mouse skin samples of ACF-treated group and control group. **(A)** The expression of antioxidant enzyme SOD-1 was detected in both the ACF-treated and control groups at each time point. **(B)** Quantitative reverse transcription PCR analysis of the SOD-1 expression in the dermis of both groups. The expression of SOD-1 was higher in the ACF-treated group than in the control group at weeks 2–4. P (week 1) = 0.707, F (week 1) = 0.164; P (week 2) = 5.283E-05, F (week 2) = 0.747; P (week 4) = 1.253E-06, F (week 4) = 0.228. Scale bar = 100 μm.

**FIGURE 13:**
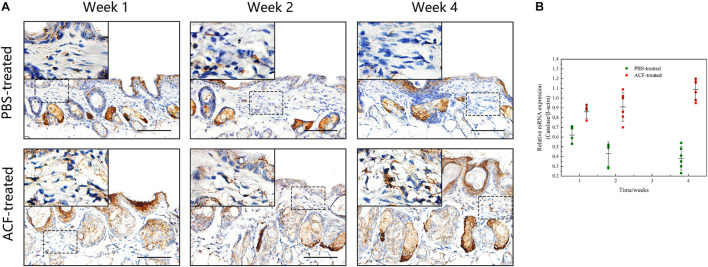
The expression of catalase of mouse skin samples of ACF-treated group and control group. **(A)** The expression of antioxidant enzyme catalase was detected in both the ACF-treated and control groups at each time point. **(B)** Quantitative reverse transcription PCR analysis of the catalase expression in the dermis of both groups. The expression of catalase was higher in the ACF-treated group than in the control group at all time points. P (week 1) = 4.278E-04, F (week 1) = 0.639; P (week 2) = 9.229E-05, F (week 2) = 0.600; P (week 4) = 6.596E-07, F (week 4) = 0.527. Scale bar = 100 μm.

**FIGURE 14:**
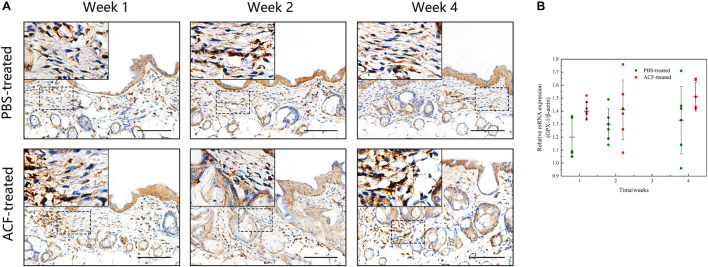
The expression of GPX-1 of mouse skin samples of ACF-treated group and control group. **(A)** The expression of antioxidant enzyme GPX-1 was detected in both the ACF-treated and control groups at each time point. **(B)** Quantitative reverse transcription PCR analysis of the GPX-1 expression in the dermis of both groups. The higher expression of GPX-1 was detected in the ACF-treated group than that in control groups at week 1. P (week 1) = 0.003, F (week 1) = 0.105; P (week 2) = 0.303, F (week 2) = 0.303; P (week 4) = 0.145, F (week 4) = 0.228. Scale bar = 100 μm.

Immunofluorescence staining showed that ROS production in the skin tissue was detected in both groups at all-time points (Figure 11A). Semi-quantification revealed that a higher level of ROS was observed in the control group than in the ACF-treated group at each time point (*P* < 0.05) (Figure 11B). Immunohistochemical staining showed that the expression of antioxidant enzyme SOD-1, catalase, and GPX-1 was detected in both the ACF-treated and control groups at each time point (Figures 12A, 13A, 14A). PCR evaluated the relative antioxidative gene expression of SOD-1, catalase, and GPX-1 in the mouse skin. The expression level of SOD-1 in the dermis of the ACF-treated group was higher than that in the control group at each time point (*p* < 0.05) (Figure 12B). The expression of catalase was higher in the ACF-treated group than in the control group at weeks 2–4 (*p* < 0.05) (Figure 13B). The higher expression of GPX-1 was detected in the ACF-treated than that in control groups at week 1 (*p* < 0.05) (Figure 14B).

### Adipose Collagen Fragment Promotes Angiogenesis and Reduces Cell Apoptosis in the Mouse Dermis

**FIGURE 15:**
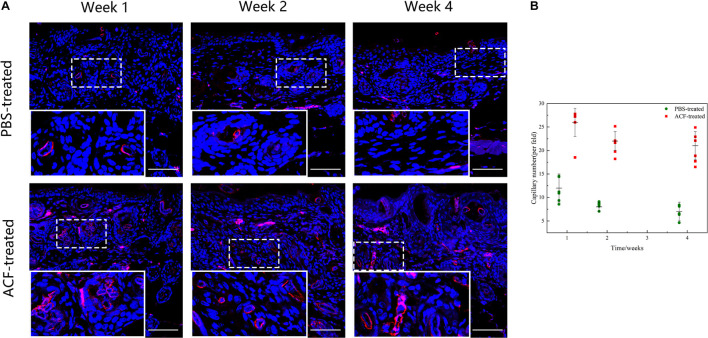
Angiogenesis in mouse skin samples of ACF-treated group and control group. **(A)** Immunostaining of CD31 + vessels in the dermis of both groups. The expression level of neo-vessels was maximal at week 1 in both groups. **(B)** Semi-quantitative analysis of CD31 + vessels in the dermis of both groups. The number of CD31 + vessels was significantly higher in the ACF-treated group than in the control group at all-time points. P (week 1) = 1.209E-05, F (week 1) = 0.489; P (week 2) = 1.352E-07, F (week 2) = 0.022; P (week 4) = 3.817E-06, F (week 4) = 0.286. Scale bar = 200 μm.

Immunofluorescence staining showed that the expression level of neo-vessels was maximal at week 1 in both groups (Figure 15A). Semi-quantification of CD31 + neo-vessels revealed that the number of CD31 + vessels was significantly higher in the ACF-treated group than in the control group at all-time points (*p* < 0.05) (Figure 15B). TUNEL-positive cells were observed in both groups at all-time points (Supplementary Figure 2A). Semi-quantification analysis demonstrated that more TUNEL-positive cells were observed in the control group than in the ACF-treated group at each time point (*p* < 0.05) (Supplementary Figure 2B).

## Discussion

In this study, an injectable, adipokine-enriched, collagen concentrate (ACF) was produced from lipoaspirate using a fast and pure mechanical method. ACF experienced a gradual degradation process *in vivo*, and exhibited a sustained release of adipokines *in vitro* and vivo. *In vitro*, ACF prevented SA-β-gal-positive cell expression, reduced ROS production and induced antioxidant proteins expression in UVA-induced L929 cells. In mice, ACF prevented skin photoaging by stimulating collagen synthesis, increasing fibroblast expression, inducing capillary formation, promoting antioxidant action, and attenuating cell apoptosis.

Sustain release systems are considered a superior approach for enhancing growth factor-based therapies with a short release duration, which requires repeated dosing (Davoodi et al., 2018). In this study, immunohistochemistry staining and a mass spectrometry analysis showed that ACF is an adipose ECM concentrate containing a large number of adipokines. The ECM is a highly specialized three-dimensional network consisting of collagen scaffolds and scaffold-bound bioactive components (Mecham, 2012). ECM binding sites, such as GAGs and proteoglycans, can bind to and sequester bioactive components (Witjas et al., 2019) as well as maintain their activity and stability (Sutherland, 2001). A release profile analysis showed a sustained release of adipokines from ACF *in vitro*. Western blot analysis which used antibodies that can only detect human-derived proteins indicated a sustained release of adipokines from ACF implants *in vivo*. Matrix-tracing technique was used to label the collagen components of ACF by using NHS ester (Cheung et al., 2014; Yu et al., 2017; Jeon et al., 2020) and our fluorescence tracing results indicated a gradual degradation process of ACF *in vivo*. Notably, the ECM serves as a natural reservoir of growth factors and releases them when exposed to the appropriate stimuli (Martino et al., 2015; Ma et al., 2020). The mechanisms that regulate the release of ECM-bound growth factors are complex, including binding affinity, conformational changes, and degradation of the ECM (Sternlicht and Werb, 2001; Afratis et al., 2018). Therefore, ACF may be considered as a sustained release system of adipokines and thus partly explain the therapeutic effects of ACF. In the past decade, several artificial sustained-release scaffolds made of synthetic materials have been developed to prevent skin photoaging (Garcia-Gonzalez et al., 2009; Fan et al., 2019; de Oliveira et al., 2020). Although artificial scaffolds can prolong the effective drug release duration, hydrophilia deficiencies and a lack of bioactivity may compromise the ability of artificial scaffolds to facilitate material-host interactions. Furthermore, their degradation products may generate an acidic environment conducive to an adverse inflammatory response and considerable cytotoxicity (Hussey et al., 2018; Wang et al., 2021).

Skin photoaging is characterized by a combination of histological findings, including decreased dermis thickness, increased collagen fragmentation, increased oxidative stress, and increased inflammatory reactions (Gu et al., 2020). Therefore, a comprehensive therapeutic strategy that ameliorates multiple interrelated manifestations of photoaging is highly desirable. GO analysis showed that the adipokines found in ACF, such as adiponectin, VEGF, FGF, transforming growth factor-β, endothelial growth factor, and hepatocyte growth factor, are involved in various biological processes. The therapeutic effects of these adipokines have been investigated in several studies. For instance, adiponectin exerts an antioxidant effect on fibroblasts by decreasing basal matrix metalloproteinase-1 expression and inducing the expression of procollagen (Kim et al., 2016). VEGF-B protects retinal cells against oxidative stress and rescues retinal degeneration by upregulating the antioxidative genes GPX1 and SOD1 (Arjunan et al., 2018). Hepatocyte growth factor protects mesenchymal stem cells against H_2_O_2_-induced apoptosis by decreasing the phosphorylation of extracellular signal-regulated kinases and p38 (Choi et al., 2019). In this study, the therapeutic effects of ACF on photoaging are likely due to an attenuation of oxidative stress levels, an improvement in fibroblast viability, stimulation of collagen synthesis, inhibition of apoptosis, and stimulation of blood vessel formation. Importantly, the comprehensive therapeutic effects of ACF may be explained by the presence of a diversity of adipokines. Nevertheless, the mechanisms underlying adipokines therapeutic effects in ACF need to be investigated in the future. Proteomic analysis showed that the extracellular space proteins represent only about 20% of all identified proteins in ACF, therefore, there are possible effects by the other proteins in preventing skin photoaging. The antioxidative effects of these proteins have been researched in several studies. For instance, nuclear protein SIRT1 is known to deacetylate FOXO3a, which has been found to induce antioxidant responses via modulation in SOD2 (Brunet et al., 2004), and cytoplasmic protein SIRT2 deacetylate PGC−1α, which thereby modulates mitochondrial biogenesis and has been associated with reduction in ROS levels and upregulation of antioxidant enzyme expression (Singh et al., 2018; Wang et al., 2020). Hence, it is important to investigate possible involvement of other proteins in the ACF mixture in skin photoaging.

In the past decade, several adipose-derived products, such as microfat and SVF-gel, were developed and considered to exert a therapeutic effect on the skin (Xu et al., 2018; Yang et al., 2021). However, limited by the particle size, intradermal injection of these adipose derivatives may be largely limited (Tonnard et al., 2013; Cohen et al., 2017; Suh et al., 2019). In addition, the fat particles and lipids contained in these adipose derivatives may cause yellowish discoloration and nodules after a superficial subcutaneous injection. Compared with adipose derivative therapies and stem cell-based therapy, the advantages of using ACF can be summed up as follows: (1) ACF is adipose collagen fragments without fat particles and lipids, and therefore does not cause yellowish discoloration and nodules after intradermal injection. (2) ACF is a viable cell-free production and therefore can overcome the challenges of stem cell-based therapies, for example, healthcare regulatory issues, poor survival of administered cells, and the risk of biological contamination. (3) ACF is obtained from lipoaspirate through a fast and pure mechanical method without chemical and biological contamination, largely reducing any potential safety hazards. (4) Prepared using simple and rapid methods, ACF can be obtained in operating rooms during surgery, largely reducing the human and economic burden. (5) Current strategies for preventing skin photoaging are mainly focused on stimulating neocollagen synthesis in the dermis (Lee et al., 2016; Chung et al., 2019; Hu et al., 2019). In comparison, ACF, as the adipose-derived collagen concentrate, can directly replenish dermal collagen components and simultaneously stimulate neocollagen synthesis in the dermis. (6) Skin aging is an inevitable and continuous physiological process (Gu et al., 2020) that requires repeated treatments to maintain the desired therapeutic effect. Lyophilization is a convenient, safe, and cost-effective method for preserving biological components (Nail et al., 2002). Lyophilized ACF for repeated future use will be investigated in the following studies. In spite of these advantages, there are still some limitations that remain to be improved. First, massive dead cells and intracellular debris (DNA/RNA) were observed in the ACF. It has been proven that the residual cellular and intracellular debris can lead to undesired host reactions, consequently resulting in adverse calcification (Zhang et al., 2010; Kawecki et al., 2018). Thus, an effort should be taken for residual DNA and RNA extraction. The optimal technique should assume the proper use of a non-toxic cell removal agent and detergent to avoid the presence of calcification. Second, the proteomic analysis showed that amass of the quantified proteins of ACF were from the cell membrane. Since the immune response is primarily directed against proteins of the cell membrane, the risks of these components should be explored in future investigations (Sullivan et al., 2012). Future clinical applications will focus on autologous applications, so the impacts and risks of calcification will be greatly reduced. Third, the optimum therapeutic concentration and dose of ACF remain unclear and should be investigated. Fourth, the duration of *in vivo* degradation of ACF needs to be assessed to help determine the optimal therapeutic schedule. Fifth, the therapeutic effects of ACF on skin photoaging in clinical settings needs to be verified. Last, as an adipokine reservoir, the therapeutic effects of ACF on skin disorders, such as melasma and atopic dermatitis, are unknown and should be determined. Fifth, the adipokines release pattern of ACF was only studied using an *in vitro* experiment due to the limitations and the *in vivo* release pattern is worthy of future investigation.

## Conclusion

ACF is an adipokines-enriched, sustained-release ECM collagen scaffold that exhibits a significantly higher therapeutic effect on mouse skin photoaging through enhancing angiogenesis, antioxidant abilities, antiapoptotic activities, and collagen synthesis. ACF may serve as a novel autologous skin filler for skin rejuvenation applications in the clinic.

## Data Availability Statement

The original contributions presented in the study are included in the article/Supplementary Material, further inquiries can be directed to the corresponding author/s.

## Ethics Statement

The studies involving human participants were reviewed and approved by the Nanfang Hospital Ethics Committee. The patients/participants provided their written informed consent to participate in this study. The animal study was reviewed and approved by the Nanfang Hospital Institutional Animal Care and Use Committee.

## Author Contributions

JG contributed to the conception and design. YH and YY contributed to the design and helped with the manuscript writing. XJ contributed to the data analysis and the manuscript writing. YZ helped with the *in vivo* experiments. XZ helped with the *in vitro* experiments and data analysis. YL, MX, KL, JR, and CM helped with the interpretation and data collection. All authors read and approved the final manuscript.

## Conflict of Interest

The authors declare that the research was conducted in the absence of any commercial or financial relationships that could be construed as a potential conflict of interest.

## Publisher’s Note

All claims expressed in this article are solely those of the authors and do not necessarily represent those of their affiliated organizations, or those of the publisher, the editors and the reviewers. Any product that may be evaluated in this article, or claim that may be made by its manufacturer, is not guaranteed or endorsed by the publisher.

## Footnotes

1
http://www.geneontology.org

2
https://www.qiagenbioinformatics.com

## Supplementary Material

Supplementary Figure 1 The ACF extractor and the unidirectional filter. The ACF extractor consists of a machine unit (idle speed = 30,000 rpm) and a sealed container (250 ml), which has a revolving disc fitted with 2 steel blades (length = 6 cm).

Supplementary Figure 2 Cell apoptosis in mouse skin samples of ACF-treated group. **(A)** Immunostaining of apoptotic cells following labeling with a TUNEL staining kit in the dermis of both groups. TUNEL-positive cells were observed in both groups at all-time points. **(B)** Semi-quantitative analysis of apoptotic cells in the dermis of both groups. More TUNEL-positive cells were observed in the control group than in the ACF-treated group at each time point. P (week 1) = 5.453E-09, F (week 1) = 0.508; P (week 2) = 1.362E-07, F (week 2) = 0.178; P (week 4) = 1.220E-07, F (week 4) = 0.308. Scale bar = 200 μm.

Supplementary Video 1 ACF preparation procedure.

## Abbreviations

ACF: Adipose collagen fragments
ECM: Extracellular matrix
UVA: Ultraviolet A
ROS: Reactive oxygen species
SVF: Stromal vascular fraction
PBS: Phosphate buffered saline
LFQ: Label-free quantification
GO: Gene ontology
FGF: Fibroblast growth factor
VEGF: Vascular endothelial growth factor
SA-β-gal: Senescence-associated β-galactosidase
GPX-1: Glutathione peroxidase 1
SOD-1: Superoxide dismutase-1.
