# Supplementary figures and images for: An Adipose-Derived Injectable Sustained-Release Collagen Scaffold of Adipokines Prepared Through a Fast Mechanical Processing Technique for Preventing Skin Photoaging in Mice
Source: Front Cell Dev Biol. 2021 Sep 24;9:722427. doi: 10.3389/fcell.2021.722427 (PMC8497903; doi:10.3389/fcell.2021.722427)

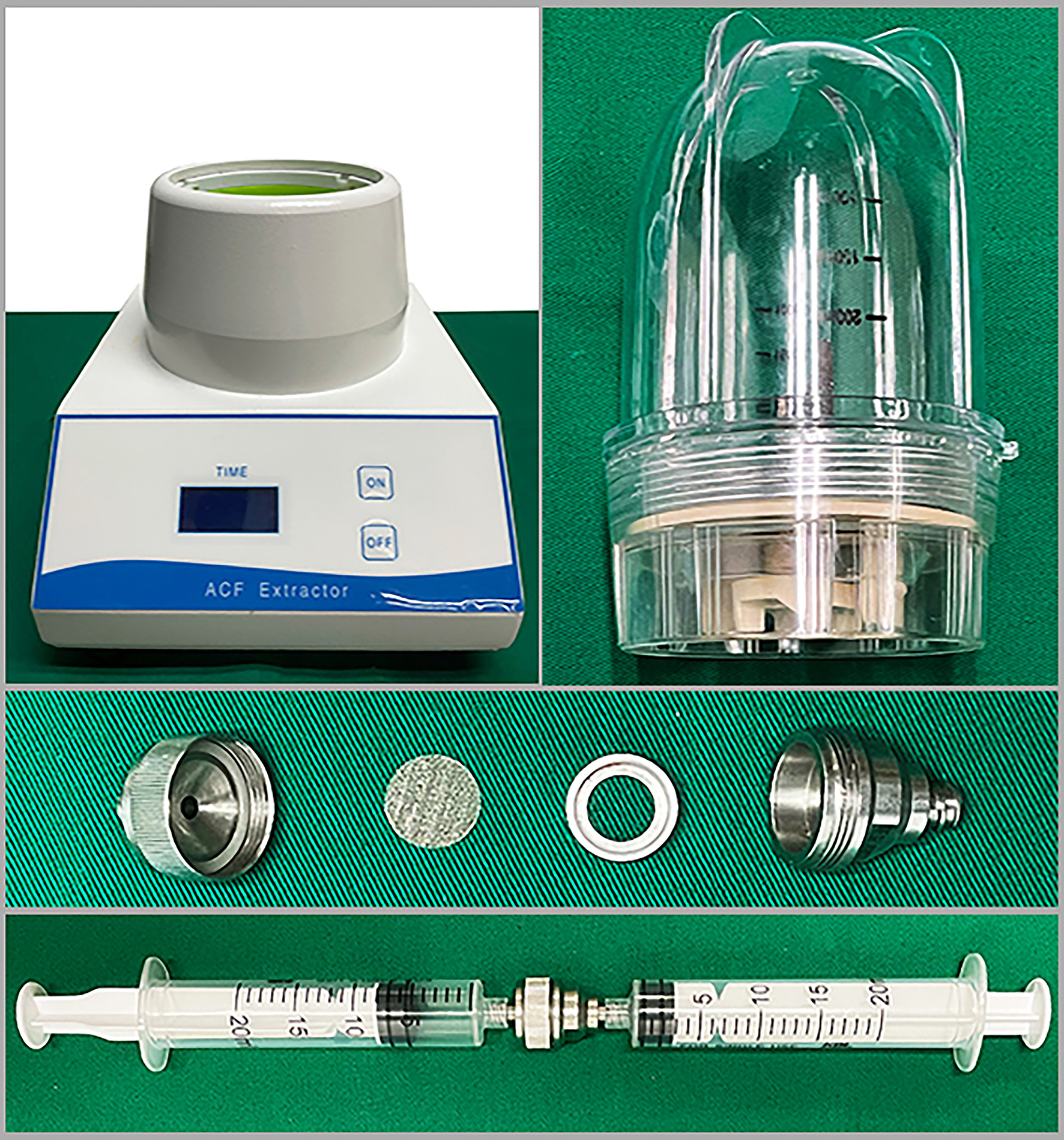

Supplement: Supplementary Figure 1 — The ACF extractor and the unidirectional filter. The ACF extractor consists of a machine unit (idle speed = 30,000 rpm) and a sealed container (250 ml), which has a revolving disc fitted with 2 steel blades (length = 6 cm). [file Image_1.JPEG]

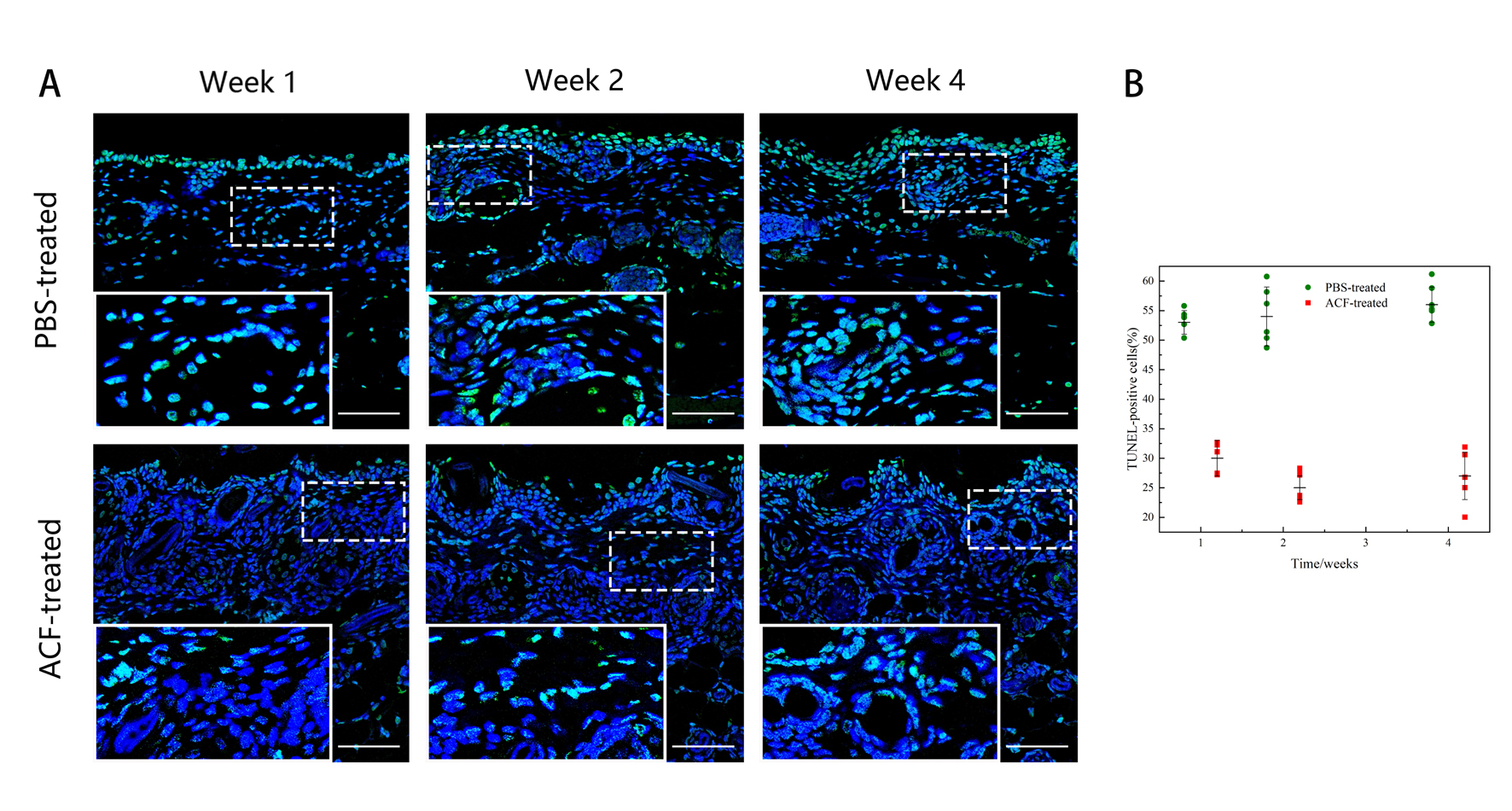

Supplement: Supplementary Figure 2 — Cell apoptosis in mouse skin samples of ACF-treated group. (A) Immunostaining of apoptotic cells following labeling with a TUNEL staining kit in the dermis of both groups. TUNEL-positive cells were observed in both groups at all-time points. (B) Semi-quantitative analysis of apoptotic cells in the dermis of both groups. More TUNEL-positive cells were observed in the control group than in the ACF-treated group at each time point. P (week 1) = 5.453E-09, F (week 1) = 0.508; P (week 2) = 1.362E-07, F (week 2) = 0.178; P (week 4) = 1.220E-07, F (week 4) = 0.308. Scale bar = 200 μm. [file Image_2.JPEG]
