# Supplementary material for: An Adipose-Derived Injectable Sustained-Release Collagen Scaffold of Adipokines Prepared Through a Fast Mechanical Processing Technique for Preventing Skin Photoaging in Mice
Source: Front Cell Dev Biol. 2021 Sep 24;9:722427. doi: 10.3389/fcell.2021.722427 (PMC8497903; doi:10.3389/fcell.2021.722427)
Supplement: Supplementary file 4 [file Table_1.DOCX]

**Supplementary Table 1**. Distribution of proteins related to angiogenesis and antioxidant ability.

| **Angiogenesis-related proteins** | **Gene** | **antioxidant-related proteins** | **Gene** |
| --- | --- | --- | --- |
| 1-phosphatidylinositol 4,5-bisphosphate phosphodiesterase delta-1 | PLCD1 | Superoxide dismutase [Cu-Zn] | SOD1 |
| Laminin subunit alpha-5 | LAMA5 | Hepatocyte growth factor | HGF |
| Protein S100-A6 | S100A6 | Catalase | CAT |
| Protein S100-A13 | S100-A13 | Adiponetin | ADIPOQ |
| Vascular endothelial growth factor | VEGF | Fibroblast growth factor 2 | FGF2 |
| Transforming growth factor beta-1 proprotein | TGFB1 | Superoxide dismutase [Mn], mitochondrial | SOD2 |
| Protein S100-A4 | S100-A4 | Glutathione peroxidase 1 | GPX1 |
| Protein S100-A11 | S100-A11 | Haptoglobin | HP |
|  |  | Peroxiredoxin-6 | PRDX6 |
|  |  | Peroxiredoxin-2 | PRDX2 |
|  |  | Extracellular superoxide dismutase | SOD3 |
