# Supplementary material for: An Adipose-Derived Injectable Sustained-Release Collagen Scaffold of Adipokines Prepared Through a Fast Mechanical Processing Technique for Preventing Skin Photoaging in Mice
Source: Front Cell Dev Biol. 2021 Sep 24;9:722427. doi: 10.3389/fcell.2021.722427 (PMC8497903; doi:10.3389/fcell.2021.722427)
Supplement: Supplementary file 5 [file Table_2.DOCX]

**Supplementary Table 2**. Distribution of proteins related to cell proliferation, and apoptosis.

| **Cell proliferation-related proteins** | **Gene** | **Antiapoptosis-related proteins** | **Gene** |
| --- | --- | --- | --- |
| Antigen peptide transporter 2 | TAP2 | Parkinson disease protein 7 | PARK7 |
| Serotransferrin | TF | Astrocytic phosphoprotein PEA-15 | PEA15 |
| Laminin subunit alpha-5 | LAMA5 | Peroxiredoxin-6 | PRDX6 |
| Phospholipid phosphatase 1 | PLPP1 | Peptidyl-prolyl cis-trans isomerase A | PPIA |
| Fatty acid-binding protein | FABP4 | Peroxiredoxin-2 | PRDX2 |
| Purine nucleoside phosphorylase | PNP | Superoxide dismutase | SOD1 |
| Bone marrow stromal antigen 2 | BST2 | Reticulophagy regulator 3 | RETREG3 |
| Eukaryotic translation initiation factor 5A-1 | EIF5A | Receptor-type tyrosine-protein phosphatase gamma | PTPRG |
| Transgelin-2 | TAGLN2 | Rho GDP-dissociation inhibitor 2 | ARHGDIB |
| 1-phosphatidylinositol 4,5-bisphosphate phosphodiesterase delta-1 | PLCD1 | Sorbin and SH3 domain-containing protein 1 | SORBS1 |
| Serine/threonine-protein phosphatase PP1-alpha catalytic subunit | PPP1CA | Actin, aortic smooth muscle | ACTA2 |
| SUMO-conjugating enzyme UBC9 | UBE2I | Gamma-synuclein | SNCG |
| Peroxiredoxin-2 | PRDX2 | Lactoylglutathione lyase | GLO1 |
| Transgelin-2 | TAGLN2 |  |  |
| Claudin-5 | CLDN5 |  |  |
| Ferritin light chain | FTL |  |  |
| Receptor-type tyrosine-protein phosphatase gamma | PTPRG |  |  |
| Fibrillin-1 | FBN1 |  |  |
